# The Hypoxostat Model: A Conceptual Framework Linking Hypoxia, Oxidative Stress and Periodontal Breakdown Under Orthodontic Load

**DOI:** 10.3390/antiox15030363

**Published:** 2026-03-12

**Authors:** Anna Ewa Kuc, Paulina Kuc, Laurentia Schuster, Michał Sarul

**Affiliations:** 1 Department of Dentofacial Orthopedics and Orthodontics, Wroclaw Medical University, 50-425 Wroclaw, Poland; 2 Faculty of Medicine, Medical University in Bialystok, ul. Kilińskiego 1, 15-089 Białystok, Poland; paulinakucc@wp.pl; 3 Department of Periodontology and Operative Dentistry, University of Münster, 48149 Münster, Germany; laurentia.schuster@ukmuenster.de; 4 Department of Integrated Dentistry, Wroclaw Medical University, 50-425 Wroclaw, Poland; michal.sarul@umw.edu.pl

**Keywords:** bone remodeling, hypoxia, HIF-1α signaling, oxidative stress, reactive oxygen species (ROS), reperfusion injury, tissue perfusion, osteoclastogenesis, redox biology, periodontal ligament

## Abstract

Background: Hypoxic and oxidative stress states tightly regulate bone and periodontal remodeling, yet the field lacks an integrated conceptual framework explaining how fluctuating oxygen availability and redox signaling determine anabolic versus catabolic outcomes. Although hypoxia-inducible factor-1α (HIF-1α), reactive oxygen species (ROS), and reperfusion injury are individually well-studied, their coordinated role in defining tissue remodeling thresholds remains unclear. Methods: This Perspective synthesizes mechanistic evidence from cellular, molecular, and tissue-level studies on hypoxia, redox biology, perfusion dynamics, osteoimmunology, and bone remodeling. Published data were evaluated to characterize how oxygen tension, ROS generation, and inflammatory signaling interact under mechanical or metabolic stress. A conceptual model (“Hypoxostat Model”) was constructed to describe the regulatory balance between hypoxia-driven catabolism and oxygenation-driven anabolism. Hypothesis: The Hypoxostat Model proposes that tissues operate within a dynamic oxygen-dependent regulatory window. Moderate hypoxia transiently activates HIF-1α, angiogenesis, and osteogenic compensation, whereas deeper or sustained hypoxia collapses perfusion, increases ROS, amplifies IL-1β/TNF-α/IL-17A signaling, and promotes RANKL-mediated osteoclastogenesis. Reoxygenation phases trigger additional oxidative bursts, further biasing tissues toward destructive remodeling. Thin periodontal phenotypes exhibit reduced perfusion reserve and increased sensitivity to hypoxia–ROS transitions, lowering their threshold for entry into catabolic remodeling domains. Conclusions: Hypoxia and redox signaling function as a bistable regulatory system controlling bone and periodontal remodeling. The Hypoxostat Model provides a unifying framework linking oxygen tension, ROS dynamics, inflammatory cytokines, and remodeling outcomes. Recognizing hypoxia–reoxygenation behavior as a mechanistic switch may improve prediction of tissue vulnerability and guide therapeutic strategies aimed at modulating redox balance or enhancing local perfusion.

## 1. Introduction

The periodontal ligament (PDL) is a highly vascularized and mechanosensitive connective tissue whose biological response to orthodontic force depends critically on microvascular integrity and oxygen availability. Under compressive loading, rapid collapse of PDL venules and capillaries produces localized hypoxia, stabilizes hypoxia-inducible factor-1α (HIF-1α), and alters cellular metabolism [1,2,3]. One immediate consequence of oxygen deprivation is the mitochondrial overproduction of reactive oxygen species (ROS), which function not only as signaling molecules but also as amplifiers of inflammatory and catabolic pathways within periodontal tissues [4,5,6].

While the classical view of orthodontic biology interprets tissue outcomes primarily through compression–tension asymmetry and strain magnitude, accumulating evidence suggests that oxygen tension and oxidative stress may represent a more fundamental regulatory axis governing periodontal adaptation and degeneration [7,8,9]. Mild or transient hypoxia can stimulate angiogenesis and soft-tissue repair through HIF-1α–driven VEGF signaling [1,10], whereas sustained or severe hypoxia increases ROS accumulation, activates RANKL-mediated osteoclastogenesis, promotes collagen degradation, and accelerates bone resorption [4,5,11].

Importantly, the thin periodontal phenotype exhibits reduced vascular reserve, diminished extracellular matrix buffering capacity and higher susceptibility to ischemic injury, leading to disproportionately severe hypoxic and oxidative stress responses even under relatively modest mechanical loads [12,13,14]. These characteristics may explain why two patients exposed to similar orthodontic forces can experience vastly different biological outcomes.

Recent integrative mechanobiological work supported by finite element modeling (FEM) and proof-of-concept CBCT suggests that tension-dominant loading patterns can preserve the buccal periodontal phenotype, whereas unfavorable loading distributions may increase susceptibility to breakdown. These observations motivate a formal “oxygen–hypoxia window” framework in which mechanical configuration governs the likelihood of transitioning from adaptive hypoxia to pathological hypoxia [15,16].

Despite extensive research on periodontal inflammation, oxidative stress and angiogenic signaling, the field lacks a unified conceptual framework integrating oxygen tension, ROS thresholds and tissue susceptibility into a mechanobiological model. In this perspective, we propose the *Hypoxostat Model*—a three-window framework describing how physiological, adaptive, and pathological hypoxia determine periodontal fate under orthodontic loading. By synthesizing established findings from vascular physiology, oxidative stress biology, mechanotransduction and phenotype research, this model aims to clarify the mechanisms underlying periodontal safety and individual variability during orthodontic treatment. This article presents a conceptual framework synthesizing current mechanistic evidence; it does not report new experimental data. This work focuses on hypoxia as a mechanically induced microenvironmental state, without attempting to integrate strain-rate–dependent or higher-order mechanobiological frameworks.

Unlike traditional force-centered paradigms that interpret periodontal breakdown primarily as a function of load magnitude, the Hypoxostat model introduces fluctuating oxygen availability as a primary regulatory variable. The novelty of this framework lies in conceptualizing hypoxia not as a binary state but as a dynamic, time-dependent signal that interacts with ROS amplification and redox-dependent inflammatory signaling. By positioning oxygen fluctuation and reoxygenation dynamics at the center of mechanobiological regulation, the model provides an integrative explanation for phenotype-dependent variability in periodontal remodeling under comparable mechanical loads.

## 2. Microvascular Physiology of the Periodontal Ligament

The periodontal ligament (PDL) contains one of the most densely perfused microvascular networks in the craniofacial system, supporting high cellular turnover and rapid metabolic adaptation. The vasculature is composed of fine arterioles, capillary loops, and venules that run parallel to collagen fiber bundles, forming a structure exquisitely sensitive to mechanical perturbation [17]. Even mild compressive force can significantly reduce local perfusion due to venular collapse, leading to a swift decline in oxygen tension within minutes of force application [18,19].

Mechanical compression produces a characteristic “ischemic gradient” in the PDL, beginning with the collapse of thin-walled venules, which are substantially more deformable than arterioles [17]. This vascular asymmetry results in rapid hypoxia in compressed regions while tensile regions preserve normal perfusion or even experience enhanced microcirculatory flow [20]. Such polarity in oxygen availability creates divergent microenvironments that prime subsequent biological responses.

Importantly, thin periodontal phenotypes exhibit reduced vascular reserve: studies have shown that individuals with thin buccal tissues present lower density of perfusing capillaries and narrower venular diameters compared with thick phenotypes [21,22]. These structural limitations lower the threshold for ischemia and intensify hypoxic and oxidative stress responses, helping explain the clinically observed susceptibility of thin phenotypes to recession, crestal bone loss, and delayed healing under orthodontic load [12,13,14].

Furthermore, microvascular compromise is not merely a passive consequence of mechanical loading but an active determinant of downstream signaling. Hypoxia-driven stabilization of HIF-1α, altered mitochondrial metabolism, and ROS generation begin precisely at the moment when perfusion drops below the adaptive window [1,2,3,4,5,6]. Therefore, understanding the vascular biomechanics of the PDL is essential for interpreting its cellular and molecular remodeling behaviors.

Together, these observations support the view that PDL microvasculature acts as the first mechanobiological sensor of orthodontic force, dictating whether local environments transition toward physiological, adaptive, or pathological hypoxia. This vascular sensitivity underpins the rationale for the proposed Hypoxostat Model, in which the magnitude, duration, and spatial distribution of ischemia define periodontal tissue fate under mechanical load.

## 3. Hypoxia Biology in Periodontal Tissues

Hypoxia is one of the earliest and most influential biological consequences of orthodontic compression. When local oxygen tension drops, periodontal ligament (PDL) cells rapidly stabilize hypoxia-inducible factor-1α (HIF-1α), a transcription factor that reprograms cellular metabolism, angiogenesis, and inflammatory signaling [1,2]. In the PDL, HIF-1α accumulation shifts fibroblasts and osteoblast-lineage cells toward glycolytic metabolism, increases VEGF expression, and modulates extracellular matrix turnover [1,10,23,24,25].

Mild or transient hypoxia promotes adaptive responses. VEGF-driven angiogenesis restores perfusion and supports reparative collagen synthesis, particularly in tension-adjacent regions where intermittent oxygen fluctuations occur [10,26]. This physiological hypoxia window may contribute to controlled remodeling and wound healing following orthodontic force application.

However, when hypoxia is sustained or exceeds the adaptive threshold, its effects become detrimental. Prolonged HIF-1α signaling enhances the expression of pro-inflammatory cytokines (IL-1β, IL-6, TNF-α) and primes periodontal tissues for osteoclast recruitment [11,27]. Importantly, severe hypoxia also increases mitochondrial production of reactive oxygen species (ROS), which further amplifies inflammatory cascades, disrupts collagen integrity, and drives bone resorption [4,5,6,28,29].

Experimental data indicate that PDL cells exposed to sustained hypoxia show decreased proliferation, impaired collagen synthesis, and upregulation of matrix-degrading enzymes, suggesting that the hypoxic environment itself initiates structural weakening independent of mechanical load [23,30]. These effects are especially pronounced in thin periodontal phenotypes, where baseline oxygen supply and extracellular matrix buffering capacity are already reduced [12,13,14,21].

**Figure 1:**
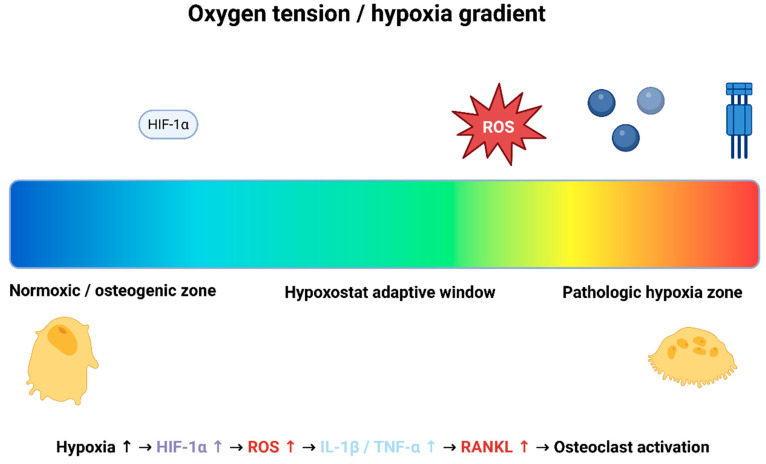
Conceptual representation of the Hypoxostat Model. Oxygen tension is shown as a continuous gradient ranging from normoxic (blue) to pathologic hypoxic conditions (red). Moderate hypoxia increases HIF-1α activity and may support compensatory or adaptive responses within a midzone “Hypoxostat window.” Deeper or sustained hypoxia leads to ROS amplification, pro-inflammatory cytokine signaling (IL-1β, TNF-α) and RANKL-mediated osteoclast activation, shifting tissues toward catabolic remodeling. Osteoblast activity predominates in normoxic/anabolic zones, while osteoclast activity dominates in high-hypoxia catabolic zones. (Created in BioRender. Kuc, A. (2026) BioRender.com/1wcr0k4).

Collectively, these findings support the concept that oxygen tension is not merely a byproduct of mechanical loading, but a central mechanobiological regulator. A drop below the physiological hypoxia range initiates a cascade involving HIF-1α stabilization, angiogenic dysregulation, ROS accumulation, and inflammatory amplification—key elements incorporated into the proposed Hypoxostat Model—Figure 1.

## 4. Reactive Oxygen Species (ROS) as an Amplifier of Tissue Damage

Reactive oxygen species (ROS) function as critical modulators of periodontal remodeling, particularly under sustained mechanical compression where oxygen supply is insufficient to maintain homeostasis. While physiological levels of ROS participate in normal signaling, excessive ROS accumulation under pathological hypoxia profoundly alters periodontal cell behavior. Mitochondrial overload, triggered by reduced oxygen availability and altered metabolic flux, leads to a surge in superoxide and hydrogen peroxide production, which reinforces and accelerates HIF-1α signaling and inflammatory gene expression [29,30,31,32].

In the periodontal ligament (PDL), high ROS levels stimulate RANKL expression in osteoblast-lineage and stromal cells, lower OPG production, and thereby shift the remodeling balance toward osteoclastogenesis [4,9,33]. This ROS-RANKL axis has been identified as a major driver of bone loss in various osteometabolic conditions and is increasingly recognized as a mechanistic link between hypoxia, inflammation, and compression-induced bone resorption. Furthermore, ROS degrade extracellular matrix components by activating matrix metalloproteinases (MMPs), oxidizing collagen fibers, and impairing fibroblast adhesion and viability [30,34].

Importantly, ROS do not act in isolation; they interact synergistically with pro-inflammatory cytokines such as IL-1β, IL-6, and TNF-α, amplifying tissue destruction through feed-forward loops [11,23]. Under sustained compressive load, this synergy produces a biochemical environment in which hypoxia, inflammation, and oxidative stress reinforce one another. Excess ROS also impair angiogenic recovery by destabilizing endothelial cells and inhibiting VEGF-dependent capillary formation [1,10,35], limiting the tissue’s ability to exit the hypoxic state.

In addition to sustained hypoxia-driven ROS production, repeated cycles of compression and reoxygenation may generate oxidative bursts analogous to ischemia–reperfusion injury observed in other vascularized tissues. During reoxygenation phases, mitochondrial electron transport chain instability can produce transient but amplified ROS spikes, further enhancing inflammatory signaling and RANKL expression. Such hypoxia–reoxygenation oscillations may therefore represent a critical mechanism by which intermittent orthodontic loading exacerbates oxidative stress beyond the hypoxic phase itself.

Collectively, these findings indicate that ROS serve as a central “amplifier” in the periodontal response to mechanical stress, converting adaptive hypoxia into a destructive cascade when oxidative thresholds are exceeded. This amplification mechanism is especially relevant in thin periodontal phenotypes, where reduced vascularity and ECM buffering accelerate ROS accumulation, lowering the threshold for irreversible tissue breakdown [12,13,14,21].

## 5. The Hypoxostat Model: A Three-Window Framework for Periodontal Response to Mechanical Load

Mechanical loading generates a spectrum of oxygen tensions within the periodontal ligament (PDL), and accumulating evidence suggests that these oxygen-dependent states, rather than force magnitude alone, determine whether tissues adapt or degenerate. Based on vascular physiology, oxidative stress biology, and periodontal remodeling research, we propose the Hypoxostat Model, a three-window conceptual framework describing how graded hypoxia and reactive oxygen species (ROS) thresholds regulate tissue fate under orthodontic load.

Window I—Physiological Hypoxia

Physiological hypoxia occurs during mild or transient mechanical compression, where temporary venular collapse reduces oxygen tension but does not surpass cellular adaptive capacity. In this zone, HIF-1α activation is modest and primarily stimulates VEGF-driven angiogenesis and endothelial survival [1,10,26]. Periodontal fibroblasts increase glycolytic metabolism while maintaining collagen synthesis, and ROS levels remain low and tightly regulated [28]. This environment supports tissue preservation, facilitates restoration of perfusion, and promotes controlled remodeling. Physiological hypoxia thus represents a protective loading window.

Window II—Adaptive Hypoxia

Adaptive hypoxia emerges under moderate, sustained compressive load. Oxygen tension falls sufficiently to increase HIF-1α stabilization and metabolic reprogramming, yet ROS production remains within the reparative range [23,30]. In this zone, the RANKL/OPG ratio shifts slightly toward osteoclast activation, permitting controlled bone resorption while VEGF maintains angiogenic support [27,36,37,38]. ROS act as necessary second messengers, enhancing remodeling without causing irreversible matrix damage [31]. This window reflects the tissue’s physiological capacity for balanced adaptation—remodeling proceeds, but structural integrity is preserved.

Window III—Pathological Hypoxia

When mechanical compression exceeds vascular and metabolic resilience, the system transitions into pathological hypoxia. Severe and prolonged oxygen deprivation causes excessive ROS production, mitochondrial dysfunction, endothelial destabilization, and a sharp increase in inflammatory cytokines [4,11,28,33]. High ROS concentrations oxidize collagen, activate MMPs, impair fibroblast adhesion, and amplify RANKL-mediated osteoclastogenesis [34,35,36,39]. Angiogenic recovery is suppressed, trapping tissues in a sustained hypoxic–inflammatory loop that accelerates PDL necrosis, hyalinization, and crestal bone loss [35,40]. Thin periodontal phenotypes enter this destructive window at substantially lower mechanical thresholds due to reduced vascular reserve and ECM buffering capacity [12,13,14,21].

The transition from physiological to pathological hypoxia is non-linear and reflects the interplay between perfusion collapse, ROS accumulation, and immune activation. The Hypoxostat Model posits that periodontal tissues possess definable oxygen and ROS thresholds that dictate whether they operate in a regenerative, adaptive, or destructive mode. This framing provides a mechanistic explanation for patient-specific variability—why identical forces can be well tolerated in one individual but lead to recession or dehiscence in another.

By centering oxygen tension and ROS thresholds as the primary regulators of periodontal remodeling, the Hypoxostat Model reframes orthodontic risk assessment around vascular competence, phenotype, and oxidative resilience. It suggests that optimizing perfusion, minimizing sustained compression, and modulating oxidative stress may be essential for preventing iatrogenic periodontal damage, particularly in susceptibility-prone phenotypes.

## 6. Thin Periodontal Phenotype as a High-Hypoxia Vulnerability State

The thin periodontal phenotype is characterized by a reduced volume of supracrestal connective tissue, a narrow band of keratinized gingiva, and a thin buccal cortical plate. These structural features produce a microenvironment with limited vascular reserve, reduced extracellular matrix (ECM) buffering capacity, and lower resistance to mechanical and hypoxic stress, making thin phenotypes disproportionately susceptible to periodontal breakdown under orthodontic load [12,13,14,41].

Microvascular studies demonstrate that gingival and PDL tissues in thin phenotypes contain fewer and narrower perfusing capillaries, resulting in reduced baseline oxygen delivery compared with thick phenotypes [21,22,42,43]. This diminished perfusion capacity means that even modest compressive forces can cause venular collapse and rapid transition from physiological to pathological hypoxia. In such patients, the Hypoxostat threshold—the point at which hypoxia becomes ROS-dominant and destructive—is reached much earlier than in individuals with thicker, more resilient tissues.

The extracellular matrix in thin gingiva and PDL also provides limited mechanical damping and poor viscoelastic buffering, increasing local strain concentrations and accelerating ROS accumulation and collagen degradation [30,34,44]. Consequently, oxidative stress amplifies inflammatory cascades, elevates RANKL expression, and promotes early osteoclastic activity, contributing to crestal bone thinning and an increased risk of dehiscence [9,34].

Clinical and radiographic studies confirm that thin phenotypes demonstrate significantly higher rates of recession, buccal bone loss, and soft-tissue instability during orthodontic movement, even when light or biologically acceptable forces are used [12,13,45]. This disparity suggests that tissue susceptibility—not force magnitude—is often the decisive factor in determining periodontal outcomes.

Furthermore, thin cortical bone exhibits higher strain gradients and lower structural tolerance, making it more vulnerable to the ROS-driven osteoimmune cascade present during pathological hypoxia [14,39,46,47]. The combined effects of microvascular fragility, rapid ROS escalation and reduced ECM resilience support the concept that the thin phenotype represents a high-hypoxia-susceptible state—a biological condition predisposed to early entry into the destructive window of the Hypoxostat Model.

Recognizing this phenotype-specific vulnerability provides a mechanistic explanation for clinically observed variability in treatment outcomes and underscores the need for phenotype-based risk assessment and load modulation strategies.

## 7. Predictions of the Hypoxostat Model

The Hypoxostat Model proposes that oxygen tension and ROS accumulation act as master regulators of periodontal tissue fate under orthodontic load. By organizing periodontal biology into three hypoxia-dependent windows, the model generates several testable predictions that may explain interpatient variability and refine clinical risk assessment.

Prediction 1—Identical forces will produce different outcomes depending on vascular reserve

Patients with limited PDL perfusion or thin periodontal phenotypes will transition more rapidly from physiological to pathological hypoxia, even when force magnitude is within biologically acceptable ranges. This prediction aligns with clinical findings that recession and dehiscence occur more frequently in patients with thin tissues despite appropriate biomechanics [12,13,14,45,48].

Prediction 2—Sustained compression is more destructive than transient high-magnitude loading

Because pathological hypoxia develops from duration-dependent ischemia rather than instantaneous peak force, prolonged low-level compression may be more damaging than short, momentary high loads. Experimental studies support this by demonstrating that chronic ischemia induces ROS-mediated bone resorption and matrix degradation, whereas transient mechanical spikes do not [24,28,31,48,49].

Prediction 3—Impaired angiogenic recovery will correlate with tissue breakdown

Once tissues enter ROS-dominant hypoxia, VEGF-driven angiogenesis is suppressed, preventing revascularization and trapping tissues in a destructive loop. Imaging and histologic studies show that diminished periodontal angiogenesis correlates with hyalinization and osteoclast recruitment under orthodontic force [10,35,50,51].

Prediction 4—Oxidative buffering capacity will determine susceptibility to damage

Tissues with stronger antioxidant defenses (e.g., SOD, catalase, GPx systems) should resist ROS accumulation and remain in the adaptive hypoxia window longer. Conversely, tissues with compromised oxidative buffering—such as those in thin phenotypes or inflamed sites—will shift rapidly into pathological hypoxia [34,44,52].

Prediction 5—Redox- and perfusion-targeted interventions will shift hypoxic thresholds

The model predicts that interventions primarily aimed at reducing mitochondrial ROS bursts (e.g., antioxidant strategies, mitochondria-stabilizing approaches) and/or improving microvascular resilience and angiogenic recovery will raise the threshold for entry into ROS-dominant pathological hypoxia and reduce the risk of breakdown [53,54].

## 8. Clinical Implications

The Hypoxostat Model provides a mechanistic rationale for phenotype-based orthodontic planning by emphasizing that periodontal safety depends not solely on force magnitude, but on oxygen tension, vascular resilience, oxidative buffering, and tissue phenotype. These principles translate into several clinically relevant considerations.

First, strategies that minimize sustained compressive loading—particularly in vulnerable zones such as the mid-root and cervical third—are likely to reduce the risk of transitioning into pathological hypoxia. Evidence indicates that prolonged ischemia, rather than transient force peaks, is the principal trigger for oxidative and inflammatory cascades [28,49,55]. Load systems emphasizing shorter compression durations or more uniform strain distribution may therefore better preserve periodontal integrity.

Second, the model highlights the importance of recognizing thin periodontal phenotype as a high-risk biological condition rather than a purely anatomical variant. Patients presenting with thin gingiva, narrow keratinized zones, or reduced buccal bone thickness may benefit from modified mechanics, increased monitoring, or adjunctive vascular-supportive approaches [12,13,14,21,48,56].

Third, because ROS serve as amplifiers of the hypoxic–inflammatory axis, antioxidant or anti-inflammatory modulation may have clinical relevance. Preclinical studies show that reducing oxidative stress suppresses RANKL expression and osteoclastogenesis under mechanical challenge [53,54], suggesting a potential role for targeted biologic modulation during orthodontic treatment, particularly in phenotypically susceptible individuals.

Finally, impaired angiogenic recovery appears to be a critical determinant of irreversible tissue breakdown. Clinical and experimental observations demonstrate that insufficient VEGF-dependent revascularization correlates with hyalinization and crestal bone loss [35,51,57]. Thus, treatment protocols that avoid prolonged ischemia and preserve endothelial viability may significantly reduce iatrogenic complications.

From a clinical perspective, the Hypoxostat concept predicts that protocols biasing tissues toward tensile, better-perfused microenvironments should widen the adaptive window and reduce the probability of entering the pathological hypoxia zone—particularly in thin periodontal phenotypes with limited vascular reserve. In silico–supported models of orthodontic loading that emphasize tension dominance provide a translational rationale for this protective shift [15,16].

The Hypoxostat framework may also be integrated into postgraduate orthodontic training by reframing force planning around oxygen-aware tissue risk assessment. Residents could be trained to evaluate periodontal phenotype, vascular reserve, and duration-dependent compression patterns rather than force magnitude alone. Incorporating hypoxia–redox dynamics into case planning may support phenotype-based treatment modulation and individualized risk stratification.

Periodontal parameters relevant to Hypoxostat-based risk assessment include probing depth (PD), clinical attachment level (CAL), bleeding on probing (BOP), gingival thickness, keratinized tissue width, radiographic crestal bone level, and CBCT-derived buccal cortical thickness. These structural and inflammatory indices may serve as surrogate markers of vascular reserve and oxidative vulnerability.

Together, these implications support a shift from force-centered mechanics toward oxygen-aware, phenotype-dependent orthodontic strategies aimed at maintaining periodontal homeostasis. Integration of oxygen-aware risk stratification into clinical planning may help distinguish reversible adaptive hypoxia from emerging oxidative destabilization, thereby supporting biologically informed modulation of orthodontic force application.

## 9. Future Directions

Although the present work is conceptual, the Hypoxostat model is testable in human longitudinal settings. Prospective monitoring of gingival crevicular fluid (GCF) biomarkers—such as IL-1β, TNF-α, IL-6, RANKL/OPG ratio, 8-OHdG, total antioxidant capacity, and HIF-1α—combined with CBCT-derived cortical thickness and radiographic bone level assessment may allow identification of patients transitioning from adaptive hypoxia to ROS-dominant pathology. Such longitudinal integration of structural and redox parameters would enable empirical validation of the proposed oxygen–redox remodeling thresholds.

The Hypoxostat Model suggests several avenues for experimental and clinical investigation.

First, oxygen-sensitive biomarkers—such as HIF-1α, lactate/pyruvate ratios, or circulating oxidative stress markers—may help identify patients at risk of entering pathological hypoxia during orthodontic treatment [58]. Developing non-invasive or minimally invasive tools to monitor PDL oxygenation would allow dynamic risk assessment and individualized load management.

Second, high-resolution imaging of microvascular dynamics, including contrast-enhanced ultrasound, laser Doppler flowmetry, or OCT angiography, may elucidate perfusion thresholds that precede structural damage [59,60]. These technologies could validate the model’s prediction that vascular collapse precedes ROS-driven breakdown.

Third, interventional studies targeting oxidative stress—via antioxidants, mitochondrial stabilizers, or modulation of immune mediators—could determine whether shifting hypoxic thresholds alters clinical outcomes. If pathological hypoxia is the pivotal driver of periodontal breakdown, stabilizing redox balance may prove protective, especially in thin phenotypes [52,53,54].

Forth, a dynamic mathematical model could be developed to describe oxygen fluctuation as a time-dependent variable (O_2_(t)) coupled with ROS amplitude and inflammatory signaling thresholds. Differential modeling of ischemia–reperfusion cycles may allow estimation of transition points between adaptive and pathological hypoxia windows. Such formalization would enable computational prediction of phenotype-specific breakdown risk.

Finally, the model can be extended to explore phenotype-specific biomechanics. Computational frameworks integrating vascular collapse, oxygen diffusion, and ROS kinetics may clarify how individual anatomical and biological factors shape hypoxic responses. Such multiscale models would support the development of oxygen-aware orthodontic loading protocols, reducing risk while optimizing efficiency. Inflammatory and oxidative biomarkers measurable in gingival crevicular fluid (GCF) could be integrated into a Hypoxostat-based risk algorithm. Candidate markers include IL-1β, TNF-α, IL-6, RANKL/OPG ratio, 8-OHdG, total antioxidant capacity, and HIF-1α expression. Serial monitoring of these markers during orthodontic treatment may allow early identification of patients entering the ROS-dominant pathological window.

Overall, the Hypoxostat Model provides a biologically grounded, testable scaffold for future research aimed at understanding and preventing periodontal damage during orthodontic therapy.

## 10. Conclusions

The Hypoxostat Model reframes orthodontic biology by identifying oxygen tension and oxidative stress as the primary determinants of periodontal tissue fate under mechanical load. Rather than interpreting outcomes solely through force magnitude or strain distribution, this framework emphasizes the central role of perfusion collapse, hypoxic thresholds and ROS-driven amplification in directing periodontal adaptation or destruction. The model explains why thin periodontal phenotypes enter catabolic pathways more rapidly, why identical forces can have divergent clinical consequences, and why sustained compression—not peak load—is the principal trigger of irreversible tissue breakdown.

By integrating vascular physiology, oxidative biology, mechanoinflammatory pathways and phenotype-specific susceptibility, the Hypoxostat Model provides a testable, mechanistic foundation for understanding periodontal risk during orthodontic treatment. This conceptual shift supports the development of oxygen-aware, phenotype-based loading strategies and highlights the potential of targeting hypoxia and oxidative stress to enhance treatment safety. Ultimately, recognizing hypoxia as a master regulatory axis advances both our biological understanding and our clinical approach to protecting the periodontium under orthodontic force.

## Data Availability

No new data were created or analyzed in this study. Data sharing is not applicable to this article.

## Abbreviations

PDL	periodontal ligament
ROS	reactive oxygen species
HIF-1α	hypoxia-inducible factor 1 alpha
VEGF	vascular endothelial growth factor
PD	probing depth
CAL	clinical attachment level
BOP	bleeding on probing
CBCT	cone beam computed tomography
GCF	gingival crevicular fluid
